# Nuclear organization and dynamics: The final Frontier for understanding genome regulation

**DOI:** 10.3389/fcell.2022.951875

**Published:** 2022-07-18

**Authors:** Eric C. Schirmer

**Affiliations:** Institute of Cell Biology, University of Edinburgh, Edinburgh, United Kingdom

**Keywords:** nucleus, organization, dynamics, outlook, challenges

## Introduction

The greatest remaining challenge in understanding genome regulation is to elucidate the full panoply of effects following from 3D genome organization and its temporal dynamics. Despite this field predating the discovery of transcription factors and epigenetics by 100 years, we probably know less than 10% of how 3D genome regulation works. Outstanding questions remain regarding the mechanisms underlying rapid versus post-mitotic gene repositioning, how the distinctive genome organization patterns of different tissues translates to fine-tuning of gene expression specifically in those tissues, the role of repositioning of non-gene loci such as enhancers and miRNA-encoding regions, the contributions of dynamic versus developmental genome repositioning events, the range of roles for nuclear bodies, and the interplay between genome organization and other factors such as epigenetics, nuclear transport, and mechanics to name a few. Major technical challenges range from understanding the relationship between chromosome connections to nuclear structures and the mechanics and physical forces that follow to quantitative aspects such as distinguishing how much genome organization or epigenetics each contribute to gene expression or determining the extent of phase separation that actually occurs in cells. The goal of the new Section on Nuclear Organization and Dynamics is to not only highlight such studies, but also provide a platform to help the field identify best practices and uniform standards so that it is easier to compare the results of different studies.

The field of genome organization effectively started when microscope advances in the 1830s allowed resolution to 1 µm (Lister, 1830) and thus first began to be able to distinguish nuclear features including the nucleoli (Wagner, 1835; Valentin, 1836). Importantly, visible changes in the structure and organization of the nucleus were already being equated with human disease in the 1850s, with a defining moment being the description by Sir Lionel Beale in 1860 of changes in the nuclei of cancer cells (Beale, 1860). Observations of condensed chromosomes during cell division led to seminal advances such as Rable’s 1885 (Rabl, 1885) noting the aligning of meiotic chromosomes that subsequently led to Boveri’s realizing that this could be the medium of Mendel’s observations (Mendel, 1866) that then defined chromosomes as the genetic heritable unit (Boveri, 1909). Despite this early start and the subsequent explosion of the field of genetics and the heavy usage of nuclear changes in cancer diagnosis/prognosis, the field made few advances for ∼100 years until Joe Gall’s development of nucleotide hybridization approaches (Pardue and Gall, 1969) led to being able to visualize at least highly repetitive DNA sequences such as ribosomal DNA that could be observed to accumulate around the nucleolus in 1972 (Henderson et al., 1972). Much greater progress was made in the 1980s when Thomas and Patrick Cremer began to develop tools to address the question of how the genome is organized in interphase cells leading to the discovery of interphase chromosome territories (Cremer et al., 1982; Schardin et al., 1985).

Technique development has been central to the evolution of the modern field of nuclear organization and dynamics. Refinements in the sensitivity of nucleotide hybridization approaches set the stage for observations from Harinder Singh, Amanda Fisher and others that important developmental genes reposition in a tissue-specific manner during development from the nuclear periphery to the interior as they become activated (Kosak et al., 2002; Williams et al., 2006). Global genome analysis tools such as Bas van Steensel’s DamID allowed identification of all genome regions at the nuclear periphery (Pickersgill et al., 2006) while Job Dekker’s cromosome conformation capture approach could determine all genome regions in proximity to one another (Dekker et al., 2002). These technical advances and 100 derivative or related approaches involving chromatin accessibility and DNA-protein interactions have generated so much data that they have yielded more questions than solid answers. Moreover, many of the experimental approaches used have the ability to alter the behavior of the loci investigated and most research in this area thus far has used cancer cell lines in 2D culture, which may be very different from tissues in how genome organization works.

## 5 core areas present the most pressing outstanding issues for the field

1) Cell type specific patterning vs. environmental responses. Some of the earliest observations that invigorated the modern field were of tissue-specific repositioning of important developmental genes in tissue differentiation (Brown et al., 2001; Kosak et al., 2002; Williams et al., 2006; Yao et al., 2011), but high-throughput approaches have been mostly applied to cancer cell lines and there is a dearth of information in actual tissues where 3D contacts and tension on a cell differ between tissue micorenvironments, this tension contributes to gene expression regulation, and growing cells in 2D culture systems generates significant gene expression changes from the same cells grown in 3D (Roskelley et al., 1994; Lelièvre et al., 1998). Even at the level of trying to map transcription factor binding sites, the data tend to be from a generic cancer line when binding typically varies depending on tissue-specific epigenetic marks and tissue-specific availability of heterodimer or complex partners. There is also the question of how allelic exclusion can be achieved in a tissue-specific manner and how genome organization intersects with inheritance of characteristics of maternal versus paternal chromosomes. How genome organization changes during development, between tissues, and in response to environmental changes is just the first question and once these maps have been made correctly in 4D (including time) it follows to determine what functional advantage a particular organization contributes to each tissue. A central question that has thus far been ignored is what is the efficacy level of genome organization? Not all cells in a particular tissue have the characteristic pattern of genome organization and within an individual cell not all genes manage to achieve the consensus pattern. As gene positioning defects have been implicated in several human developmental and genetic disorders, it is important to know what percentage of “optimal” genome organization yields normalcy and what yields disease. It is interesting that in muscular dystrophy and lipodystrophy recently linked to disruption of gene, enhancer, and miRNA-encoding loci positioning patterns (Robson et al., 2016; Meinke et al., 2020; Czapiewski et al., 2022), the patients develop these tissues normally until they get hit with higher metabolic loads, suggesting that a random genome organization could sustain basic tissue functions, but that the 3D genome organization optimizes tissue function to enable greater organismal capacities. In this light it would be interesting to test also atheletes where we might find that a tissue with 60–70% optimal genome organization is normal while an increase to 80% gives an athelete an olympian advantage and <40% yields disease. These questions also underscore how critical it is to determine the molecular mechanism behind establishment of these cell-type specific genome organizational patterns and its likely stochastic nature.
2) Rapid versus mitosis-dependent genome organization changes and the relative contributions of directed vs. stochastic forces. Several experimental systems indicated a requirement of cells to go through mitosis to achieve changes in genome organization (Finlan et al., 2008; Reddy et al., 2008). However, rapid genome organization changes occur in response to cell stimuli such as serum withdrawal or lymphocyte activation. Such changes require an active directed mechanism involving motors and, indeed, a role of actin and motor proteins has been indicated (Mehta et al., 2010). In development there is likely a mix of fast and slow changes and distinguishing which changes fall into each category will be necessary to interpret global changes and elucidate further details of each mechanism. To further understand how mitosis supports gene repositioning could also lead to interesting observations regarding mechanisms underlying chromatin compaction: for example, do genes that interact with mitotic vesicles from the nuclear membrane containing chromatin binding proteins have characteristics that keep them on the outside of mitotic chromosomes during the process of chromatin compaction? Another outstanding question regarding rapid repositioning events is how and under what conditions the starting positioning is restored. One argument for rapid gene repositioning changes in lymphocyte activation is that repositioning contributes to a stepwise process of basic transcriptional activation followed by enhancer-directed optimal activation and that this could be a protective mechanism for preventing cytokine shock with minor infections. A related argument is that the genome positioning changes “prime” the immune system for faster high-level activation of gene expression programs in response to subsequent challenges.
3) What tethers different chromosome regions to different nuclear bodies and the nuclear envelope and what kinds of physical forces are involved? Genes appear to be tethered to the nuclear envelope through larger complexes involving at least the intermediate filament lamin polymer, nuclear membrane proteins, transcriptional regulators, and epigenetic enzymes (Zullo et al., 2012; Demmerle et al., 2013). However, there are likely additional missing components and the nature of the mechanical forces at and on these tethers in 2D and 3D culture conditions remains a complete mystery. There is virtually no information on this for nucleolar and other nuclear body associations or how the mechanical forces are altered by breaks in chromosomes such as when DNA damage accumulates. The mechanical forces from various tethers could also be important for example in facilitating retrovirus integration or placing steric constraints on DNA: indeed differing tension on DNA using *in vitro* systems had an effect on the efficiency of retrovirus insertion and Tn3 resolvase function (Benjamin et al., 1996; Ouali et al., 1996). It is possible thus that genes close to the nuclear envelope would be stabilized by proximal lamina tethers for better binding of transcriptional regulators or more efficient unwinding of DNA by having an effective platform to more efficiently use the energy from ATP against. In fact, it remains unclear how much of the genome in a living organism might be in non-Waston-Crick canonical B-form right handed helical structures such as Z-form or any of the other letters of the alphabet which are almost used up in describing different forms of DNA (Ghosh and Bansal, 2003) and if these other forms tend to occur in particular nuclear subregions or conditions. Such physical mechanics also likely applies at a higher level with regards to the genome and/or nuclear integrity withstanding mechanical forces such as during heart contractions or stretching skin. Importantly, since DNA tension and mechanics may be transitory, it will be important to understand what mechanisms drive these changes and how they are regulated.
4) Mechanisms and relative contributions. Thus far researches have mostly just observed that an aspect of genome organization or dynamics correlates with or directs changes in gene expression: the molecular mechanisms and regulation behind these changes still need to be investigated. Moreover, it could be argued that these mechanisms “fine tune” genome regulation, but understanding for example what contribution comes from a change in DNA folding recruiting more transcriptional regulators compared to what contribution comes from generating a localized concentration of a transcription factor around a nuclear body requires development of a new set of tools. It will also be important to integrate data from many different approaches to explain poor position-function correlations for genes. While DamID studies found many genes that changed expression corresponding to their changing position, there were also many genes that repositioned without changing expression or that changed expression without repositioning. The former could be explained if genes adjacent to one another move together but only the one that also has transcriptional regulators present is altered in expression, but the latter was more inexplicable. This was made the more confusing when knockdown of nuclear membrane proteins that appear to be necessary for gene repositioning events not only blocked gene repositioning but also affected expression of many genes that did not change position (Robson et al., 2016; Gatticchi et al., 2020; Czapiewski et al., 2022). Some of this can be explained by correlations between the wider gene expression changes and transcriptional regulators, enhancers, and miRNAs encoded by non-gene genome regions that reposition in a manner dependent on these nuclear membrane proteins. How different facets within the milieu of factors contributing to genome organization combine to yield effects on one gene but not another is another important question to be tackled.
5) Lack of consistency and uniform standards across the field. As funders increasingly push for re-use of existing datasets, it has become commonplace to use a Hi-C, DamID, or ChIP-Seq dataset acquired in one tissue system to interrogate a mechanism or pathway using another tissue system where partners and behavior may differ. It is also very common to just use an available IMAGE clone cDNA when studying a protein without testing if that particular splice variant is the one expressed in the tissue/experimental systems being used. Moreover, many commonly used cancer cell lines have such a high rate of ploidy and other genome changes that labs using the “same” cell line could get differing results just because of these changes. These experimental deficiencies can yield both false-negative and false-positive results. In the currently trendy area of phase separation, different labs use different temperatures to achieve phase separation while some use molecular crowders and there are many other parameters that vary between published studies. It would be very useful to establish some common controls and standards to be run with each such study. As noted earlier, we still know very little about transcription factor targets and since most transcription factors act as heterodimers and their partners can change from tissue to tissue, we need complete transcription factor target studies for both binding and transcription in not just one cell type but all cell types for all transcription factors to model these questions to explain why only some genes and not others are affected. The 4D Nucleome venture led to great advances in technologies and identification of novel mechanisms; however, it did nothing to address the issues noted above and the vast majority of 4D Nucleome studies used cancer cell lines in 2D that are aberrant in myriad ways from ploidy to cell responses to “tissueness”. While many experimental approaches can only be performed in tissue culture, cells in culture are generally exposed to 100x more oxygen than cells in a tissue and 3D contacts and tension on a cell in the tissue environment differ enormously from 2D tissue culture resulting in differences in nuclear morphology, nuclear mechanics, and mechanosignal transduction. Such issues could in part explain historical poor correlations between gene position and function, contradictory findings between different labs, and many other problems in the field. These issues could be addressed through community changes and networks using uniform standards to map genome architecture, transcription factor binding, enhancers, epigenetics and many other factors across all tissues for humans and each major experimental organism.

Only when the issues raised above have been addressed will it be possible to establish a Grand Unified Theory of Nuclear Organization. The umbrella of nuclear and genome organization and dynamics keeps growing, including both aspects such as Epigenomics and Epigenetics, Developmental Epigenetics, Cancer Cell Biology, Stem Cell Research, and Signaling that are covered elsewhere at Frontiers in Cell and Developmental Biology and aspects that do not have their own Sections such as Nucleoskeletal and Cell Mechanics, NPC Structure and Function, and Mechanosignal Transduction. Thus, while the original intended focus for this section was on genome regulation from 3D genome organization and dynamics, the Nuclear Organization and Dynamics Section will be happy to entertain papers from this wider range of areas that currently do not have their own Section in the journal.
